# Homozygous *TNNI3* Mutations and Severe Early Onset Dilated Cardiomyopathy: Patient Report and Review of the Literature

**DOI:** 10.3390/genes14030748

**Published:** 2023-03-19

**Authors:** Ugo Sorrentino, Ilaria Gabbiato, Chiara Canciani, Davide Calosci, Chiara Rigon, Daniela Zuccarello, Matteo Cassina

**Affiliations:** 1 Clinical Genetics Unit, Department of Women’s and Children’s Health, University of Padova, 35128 Padova, Italy; ugo.sorrentino@unipd.it (U.S.); ilaria.gabbiato@studenti.unipd.it (I.G.); chiara.canciani@studenti.unipd.it (C.C.); davide.calosci@studenti.unipd.it (D.C.); chiara.rigon@unipd.it (C.R.); matteo.cassina@unipd.it (M.C.); 2 Clinical Genetics Unit, University Hospital of Padova, 35128 Padova, Italy

**Keywords:** *TNNI3*, autosomal recessive, cardiac failure, congenital cardiopathy, loss of function

## Abstract

The *TNNI3* gene encodes for the cardiac isoform of troponin I, a pivotal component of the sarcomeric structure of the myocardium. While heterozygous *TNNI3* missense mutations have long been associated with autosomal dominant hypertrophic and restrictive cardiomyopathies, the role of *TNNI3* null mutations has been more debated due to the paucity and weak characterization of reported cases and the low penetrance of heterozygous genotypes. In recent years, however, an increasing amount of evidence has validated the hypothesis that biallelic *TNNI3* null mutations cause a severe form of neonatal dilated cardiomyopathy. Here, we expand the case series reporting two unrelated patients afflicted with early onset dilated cardiomyopathy, due to homozygosity for the p.Arg98* *TNNI3* variant, which had thus far been documented only in heterozygous patients and apparently healthy carriers, and the recurrent p.Arg69Alafs*8 variant, respectively. A review of previously reported biallelic *TNNI3* loss-of-function variants and their associated cardiac phenotypes was also performed.

## 1. Introduction

Inherited cardiomyopathies (ICMs) are genetic disorders caused by defects hampering the contractile function of the cardiac muscle. Even though they represent a minority of the overall cases of cardiomyopathy [1,2], ICMs are the most prevalent form in the pediatric age group, in which they are usually associated with rapid progression and frequently fatal outcomes [3]. From a morphofunctional standpoint, cardiomyopathies are classified as dilated (DCMs), hypertrophic (HCMs), restrictive (RCMs), left ventricular noncompaction (LVNC), and arrhythmogenic, although the different phenotypes can partially overlap. In particular, DCMs are among the most common forms of ICM [4]. They are defined by the enlargement and dilation of one or both of the cardiac ventricles, resulting in a primarily systolic dysfunction. Clinical presentation can be preceded by a latent period and is often nonspecific, as the most frequent symptoms include paroxysmal dyspnea, shortness of breath, fatigue, malaise, and weakness. More severe cases can be complicated by conduction disease, arrhythmias, thromboembolic events, or even sudden cardiac death [5]. A diagnosis of DCM is primarily made on the basis of cardiac imaging (echocardiography, subsequently completed via a computed tomography scan or magnetic resonance) searching for evidence of left ventricular enlargement and impaired systolic function. The most important parameter in this regard is left ventricular ejection fraction (LVEF), which has generally been accepted as evidence of impaired systolic function when it is found to be below 40–50%. Familial DCMs specifically have also been frequently associated with additional findings, such as increased left ventricular wall thickness, prominent left ventricular trabeculations and right ventricular or atrial chamber enlargement or dysfunction [6]. On pathological examination, DCMs can be generally divided into ischemic and nonischemic subtypes [1]. Abnormal trabeculations, with characteristics compatible with LVNC, and transmural myocardial fibrosis are rather common findings; postnecrotic scars of the free wall and papillary muscle can also be found. Co-occurrence with myocarditis, often of viral origin, has also frequently been described in these patients, possibly acting in a synergistic manner with the structural defect caused by the genetic disorder [7]. When diagnosed in childhood, DMCs manifest mainly as rapidly progressive and severe diseases, as the consequent systolic and/or diastolic myocardial dysfunction ultimately results in heart failure, requiring mandatory organ transplant [8]. The majority of patients are diagnosed with heart failure within the first year of age. Evidence of familial disease is documented in approximately 15–20% of patients, transmitted for the most part in an autosomal dominant mode [9].

On the molecular level, ICMs can occur whenever a genetic mutation disrupts any of the components of the functional unit of the myocardium, i.e., the sarcomere [10], but also cytoskeletal, gap junction, and ion channel proteins that participate in cardiac function. In particular, DCMs have been recognized as the most genetically heterogeneous among ICMs, as they have been associated with more than 100 genes to date, even though the identification of a causative molecular alteration only occurs in less than half of the cases, even in familial diseases, reflecting the still insufficient knowledge of their genetic landscape [6].

The multimeric structure of the sarcomere results from the orderly assembly and the precise interaction of thick filaments, made of myosin, with thin filaments, while the giant protein titin contributes to cardiac passive tension [11]. Thin filaments are constituted by the integration of actin molecules with both tropomyosin and a Ca2+-sensitive heteromeric protein, known as troponin complex. The sarcomeric gene most frequently involved in DCM is titin (*TTN*), which accounts for up to 25% of DCM cases, although most often in older patients [12]. Genes encoding myosin proteins (*MYH6*, *MYH7* and *MYBPC3*) actin proteins (*ACTC1* and *ACTN2*), tropomyosin protein (*TPM1*), and troponin complex proteins (*TNNC1*, *TNNT2*, *TNNI3*) are instead involved in the more precocious forms of the disease [13]. The troponin complex is a regulatory system composed of three distinct subunits, namely cardiac Troponin-I (cTnI), cardiac Troponin-T (cTnT), and cardiac Troponin-C (cTnC). In particular, cTnI is known to act as an inhibitory regulator of cardiac contraction by hampering the cross-bridge interaction between actin and myosin molecules, and thus the activity of the actomyosin ATPase, in response to the reduction in intracellular calcium concentration that occurs during the diastolic phase, leading to muscular relaxation [14]. Conversely, the rise of calcium concentration during the early systolic phase activates the cTnC subunit, which in turn determines a conformational change in cTnI, causing it to no longer obstruct the actin–myosin interaction [15].

cTnI is a 24 kD protein encoded by the *TNNI3* gene, which is located on chromosome 19q13.4. De novo or inherited *TNNI3* variants have thus far been associated with a variety of pathological cardiac phenotypes. However, they demonstrate a rather consistent genotype–phenotype correlation [16,17,18,19]. Heterozygous missense mutations have been widely associated with either dilated, restrictive, hypertrophic, or intermediate cardiomyopathy phenotypes, even though the penetrance of the disease for the same mutation is characterized by extreme variability, which is both intrafamilial and interfamilial [20,21]. Healthy carriers are frequently described, although milder cases in familial DCMs are often underdiagnosed [9]. These autosomal dominant manifestations have been attributed to either gain of function or negative dominant effects of the mutated protein, disrupting its interaction with other pivotal components of the thin filaments, such as cTnC, cTnT, tropomyosin and actin [22]. As such, TNNI3 mutations associated with an autosomal dominant effect are most frequently observed in the domains responsible for the interaction with actin and cTnC, namely the inhibitory and mobile domains encoded by exon 3, 7, and 8. Conversely, the role of loss of function mutations in causing ICMs, specifically of the dilative type, has historically been less clear. In particular, heterozygous null variants in the thin filaments genes, such as *TNNI3*, have consistently been recognized as benign, to the point where heterozygous loss of function mutations in genes associated with ICM have been explicitly made a primary example of why the PVS1 criterion of the American College of Medical Genetics (ACMG) variant classification system should not be automatically attributed to null variants without a proved correlation with the underlying disease mechanism [23]. On the other hand, the possibility of DCMs inherited as an autosomal recessive trait due to biallelic loss of function mutations (DCM2A, MIM #611880), has long been speculated [24], but the supporting evidence has been comparatively scarce and poorly characterized. This association consolidated itself in recent years thanks to a sudden increase in case series reporting the identification of homozygous loss of function mutations in DCMs and RCMs patients.

In this work, we describe the clinical manifestations of two young, unrelated patients diagnosed with DCM and presenting with a homozygous genotype for the two *TNNI3* null mutations with, respectively, the highest and second-highest carrier frequencies in the general population. We also perform a review of previously reported cases of autosomal recessive *TNNI3*-related ICMs.

## 2. Materials and Methods

### 2.1. Genetic Analyses

Genomic DNA was extracted from peripheral leukocytes of probands and their parents using standard methods. Two different next-generation sequencing (NGS) technologies were used in the two probands according to the availability in our Laboratory at the time of admission: for the first patient, clinical exome sequencing was performed using the Illumina TruSight ONE Expanded kit on an Illumina NextSeq 550 system (Illumina, San Diego, CA, USA). Variants’ calling, annotation, selection and interpretation were performed as previously described [25]. For the second patient, the coding regions of a custom selection of genes were isolated and captured using the SureSelect Target Enrichment systems (Agilent Technologies, Santa Clara, CA, USA); indexed DNA fragments libraries were generated according to the manufacturer’s protocol and sequenced on a NextSeq 550 system. Variant calling, annotation, and subsequent bioinformatic analysis for Copy Number Variations were performed using the SureCall software (Agilent Technologies). Variants identified in either patient were confirmed by Sanger sequencing, following a standard protocol. Variants in the *TNNI3* gene are reported according to the RefSeq transcript NM_000363.5; the predicted change in the encoded protein is reported according to the RefSeq protein NP_001605.1. The clinical assessments and the genetic tests were part of the routine diagnostic and follow-up procedures. Written informed consent for molecular genetic studies was collected from the parents of the probands.

### 2.2. Literature Review

A retrospective review of the literature on *TNNI3* variants and DCM2A phenotype was performed. Pertinent English language articles were searched in PubMed (https://pubmed.ncbi.nlm.nih.gov/, accessed on 24 February 2023) and Web of Science (https://www.webofscience.com/, accessed on 24 February 2023) using any combination of the key words ‘*TNNI3*’, ‘CMD2A’, ‘congenital inherited dilated cardiomyopathy’, ‘autosomal recessive’, and ‘homozygous’. Demographic and genetic data, characteristics of cardiomyopathy, and accessory phenotypic features were extracted from previously reported patients. Additional pathogenic or likely pathogenic variants associated with CMD2A were searched in mutation databases, including LOVD—Leiden Open Variation Database (https://www.lovd.nl/, accessed on 24 February 2023), ClinVar (https://www.ncbi.nlm.nih.gov/clinvar/, accessed on 24 February 2023), and HGMD—Human Gene Mutation Database (http://www.hgmd.cf.ac.uk/, accessed on 24 February 2023).

## 3. Results

### 3.1. Patient A

**Figure 1:**
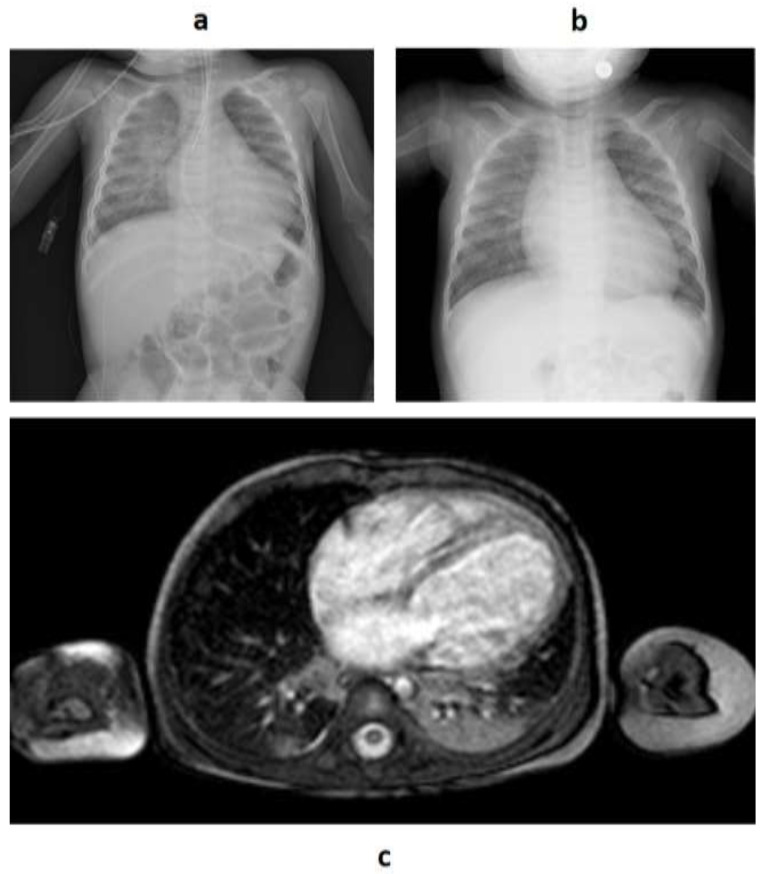
Chest X-ray imaging from patient A (**a**) and patient B (**b**) at the age of clinical onset at 7 and 6 months, respectively, showing an enlarged cardiac silhouette in both cases; (**c**) MRI transverse cardiac section of patient A, showing a markedly dilated left ventricle.

The proband was the first child of nonconsanguineous Italian parents; her family history was unremarkable in terms of cardiac or genetic diseases. She was born at full term without any prenatal or perinatal complications. Early developmental milestones and growth were reported as normal. At the age of 7 months, the child was admitted to the local emergency ward and subsequently transferred to the pediatric intensive care unit of our clinic following the onset of progressive dyspnea. Her electrocardiogram (ECG) showed sinus tachycardia and signs of biventricular overload, while transthoracic echocardiography (TTE) revealed a dilated left ventricle with thin walls and impaired systolic function with LVEF equal to 25% according to Simpson’s disk summation method; left atrial dilatation and mild mitral regurgitation were also reported, whereas the right chambers appeared normal. Virological testing documented a concomitant adenoviral infection, which initially raised the suspicion of viral myocarditis. Subsequent cardiac magnetic resonance (MRI) confirmed a dilated and markedly hypokinetic left ventricle, whose walls were particularly thinned in the inferior–lateral and middle–apical parts with discontinuous late gadolinium enhancement, in the absence of edema or myocardial fibrosis; despite the normal volume, the right ventricle was itself hypokinetic (Figure 1). A myocardial biopsy was also performed, showing nonspecific signs of lymphocytic myocarditis and endocardial fibroelastosis. The child was therefore diagnosed with dilated cardiomyopathy of uncertain etiology, as both viral and genetic causes were suspected.

Three weeks after the first admission, the patient developed acute-on-chronic heart failure and required veno-arterial extracorporeal membrane oxygenation and the insertion of a ventricular assistance device to support her cardiac function. During hospitalization, her general condition continued to worsen and major neurological complications occurred; in particular, she suffered from an ischemic stroke secondary to the occlusion of the insular segment of the right middle cerebral artery, causing her a right-sided hemiparesis. After multidisciplinary evaluation, she was listed for a heart transplant, which she received after 4 months. A significant clinical improvement was observed following the procedure. The pre-discharge echocardiography was normal, with no signs of rejection and a LVEF of 61%.

### 3.2. Patient B

Patient B was the fourth child of consanguineous (first-degree cousins) Moroccan parents. Two of his sisters were reported as healthy, while the third died of heart failure at 8 months. The pregnancy and perinatal history were unremarkable. In consideration of family history, both fetal and neonatal echocardiography were performed, without any abnormal findings. Symptomatology started insidiously with feeding difficulties, followed by sudden onset of dyspnea at the age of 6 months. A chest X-ray highlighted an enlarged cardiac silhouette, prompting further cardiologic examinations. ECG found sinus tachycardia and signs of overload of the left ventricle. TTE showed a severe dilatation of the left chambers with remarkable reduction of LVEF (25%) and mitral and tricuspid insufficiency, while the right ventricle appeared undilated with sufficient systolic function. Because of the rapid deterioration of his clinical conditions, the patient was listed for a heart transplant, which he received at the age of 8 months with favorable clinical outcome. Post-transplant pathological examination assessed dilatation of both ventricles, and microscopic evaluation found evidence of normally oriented cardiomyocytes with dysmetric proportions, extensive subendocardial cytoplasmic vacuolization from likely ischemic damage, interstitial edema, and mild fibrous thickening of the endocardium. These results were deemed compatible with DCM.

To investigate the etiology of their disease and to provide a recurrence risk to their families, both children were subjected to the molecular analysis of a selection of genes associated with cardiomyopathies.

### 3.3. Genetic Analyses

**Figure 2:**
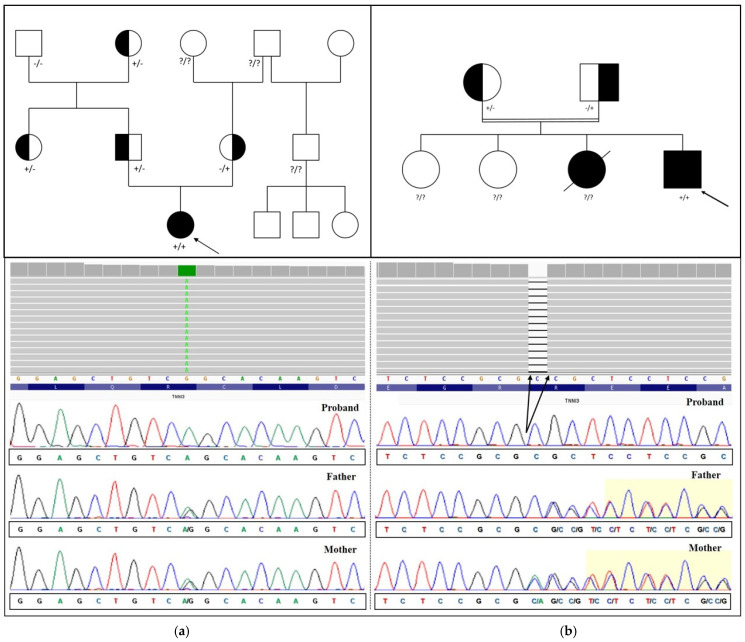
Schematic representation of family pedigrees of patient A (**a**) and B (**b**), and their respective NGS and Sanger sequencing results showing (**a**) the homozygous c.292C>T substitution identified in patient A and in her heterozygous parents, and (**b**) the c.204 deletion identified in patient B and her carrier parents. Sanger sequencing images have been captured from the reverse strand. Incidentally, the mother of patient B also proved to be compound heterozygous for the recurrent c.204G>T polymorphism.

Clinical exome sequencing analysis performed in patient A was targeted at a selection of genes associated with cardiomyopathies. The analysis identified an apparently homozygous variant in the *TNNI3* gene: NM_000363.5:c.292C>T, which is predicted to cause a premature stop codon p.(Arg98*). Segregation analysis revealed that both the parents were heterozygous carriers of the variant, confirming the homozygous genotype of the proband (Figure 2a).

The p.(Arg98*) stop codon affects exon 6 of the *TNNI3* gene. The same variant has been reported in a heterozygous patient affected by adult-onset DCM [26], and it has also been found in multiple apparently healthy heterozygous carriers according to the gnomAD 2.1 database (minor allele frequency in the non-Finnish European population = 1/9187). It has also been reported in a case of stillbirth, although without a well-defined causal correlation [27]. The main mutation databases (Clinvar, LOVD, HGMD) provided conflicting classifications, ranging from variant of unknown significance to likely pathogenic to pathogenic, and were supported by very limited clinical data. In silico pathogenicity scores were inconclusive overall. Despite the classification uncertainties regarding the previous reports in mutation databases, the identification of a rare (PM2 criterion according to ACMG classification), homozygous (PM3_supporting) nonsense variant in a gene whose loss-of-function mutations are a known mechanism of disease (PVS1 criterion) in a patient presenting with a highly compatible cardiac phenotype, should be considered strongly supportive of the variant’s pathogenic role in our proband’s disorder (class LP according to ACMG classification).

In patient B, the NGS analysis of a custom cardiomyopathy-related gene panel revealed a homozygous *TNNI3* truncating variant NM_000363.5:c.204del p.(Arg69Alafs*8) in exon 5 of the *TNNI3* gene. Allelic contribution from both parents was confirmed by segregation analysis (Figure 2b). The p.(Arg69Alafs*8) variant has already been reported in multiple unrelated cases of autosomal recessive DCMs, corroborated by functional studies performed on the heart biopsy samples from affected patients, which demonstrated markedly reduced levels of TNNI3 mRNA and the complete absence of the protein product, supporting the hypothesis of degradation by nonsense mediated decay [28]. The variant has been found in nine presumably healthy carriers in the gnomAD 2.1 database, resulting in a minor allele frequency approximately equal to 1/26,222. Thus, according to the available data, the p.(Arg69Alafs*8) variant fulfilled the PVS1 (homozygous truncating variant in the context of an established autosomal recessive disease), PS3 (functional assays proving the variant’s deleterious impact on protein function), PM2 (allele frequency below the population threshold for a recessive variant), and PM3_supporting (in trans detection of a rare variant, adjusted for homozygous occurrence in a single proband, [29]) criteria of ACMG variant classification. The homozygous mutation identified in Patient B was therefore considered pathogenic (class P ACMG).

Following the molecular diagnosis, both families were offered cardiologic evaluation at our institution. However, to our knowledge, neither set of parents performed the proposed cardiac investigations; therefore, their phenotypic characterization, beyond the anamnestic data of good general health and absence of cardiac symptoms, is incomplete. Post-test genetic counseling was also proposed and performed in the family of patient A, while the family of patient B declined the counseling; therefore, the option to extend the segregation analysis in the living, and apparently healthy, siblings of patient B could not be discussed.

## 4. Discussion

**Figure 3:**
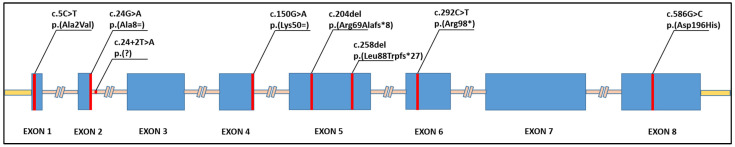
Representation of the position of reported biallelic loss-of-function mutations within the *TNNI3* gene.

The association between dilated cardiomyopathy and biallelic loss-of-function variants in the *TNNI3* gene was first suspected in the year 2004 by Murphy et al. [24], who identified two affected siblings, born from distantly consanguineous parents, carrying a homozygous alanine to valine substitution in the second residue of the gene. Their clinical presentation was compatible with the CMD2A phenotype originally described by Goldblatt et al. [30], and their family history and variant segregation were consistent with autosomal recessive transmission. However, the validity of such association was later questioned by Carballo et al. [17], who performed their own functional analysis on the p.(Ala2Val) variant, finding the effect of the substitution towards the troponin activity to be not significant. After this setback, the role of biallelic *TNNI3* mutations in DCM remained uncertain until the late 2010s, when the progressively universal implementation in the clinical setting of high-throughput parallel sequencing approaches began to provide a slow but steady increase in case series, describing cardiomyopathy phenotypes in association with homozygous or compound heterozygous *TNNI3* variants (Figure 3).

While performing a whole-exome sequencing analysis on a cohort of 42 pediatric patients, who were selected on the basis of acute myocarditis symptoms and histological evidence of inflammatory infiltrates within the myocardium, Belkaya et al. [31] found a homozygous c.150G>A p.(Lys50=) apparently synonymous *TNNI3* variant in one affected individual. The patient presented with a DCM phenotype with low LVEF (13%) and died shortly after the disease onset. In 2018 Streff et al. [32] reported a complex phenotype, characterized by the co-occurrence of early onset DCM together with Amish nemaline myopathy, in a child carrying a homozygous contiguous gene deletion of about 11 kb, which encompassed the last exon of *TNNI3*. Another biallelic variant, although in relation to a remarkably different cardiac phenotype, was published in 2019, when Pantou et al. [33] identified a homozygous p.(Asp196His) substitution in three adult siblings, two of them suffering from late onset RCM and one from HCM, while heterozygous carriers in the family were apparently healthy. Kühnisch et al. [28] further widened the phenotypic and genotypic spectrum by identifying two unrelated pediatric patients, one with early onset DCM and the other with a familial form of LVNC, carrying homozygous p.Arg69Alafs* and c.24+2T>A p.(?) truncating variants, respectively. The authors also demonstrated the histopathological effects of the reported mutations by determining the absence of TNNI3 mRNA and protein in heart biopsies of both patients, and the parallel increase in the fetal isoform TNNI1. The correlation with LVNC has been corroborated by Mehaney et al. [34], who reported the case of a 7-year-old male patient who was diagnosed at the age of 6 months with an overlapping phenotype of LVNC and DCM, and presented with a homozygous c.258del (p.Leu88Trpfs*27) mutation. The two distinct case series published in 2021 by Seidel et al. [35] and Pezzoli et al. [36] further consolidated the p.Arg69Alafs* and c.24+2T>A mutations as recurrent causes of early onset autosomal recessive DCM in four unrelated patients; notably, Seidel’s study was performed on a selected cohort of myocarditis patients, as was the case of Belkaya’s. Janin et al. [37] reported the most extensive case series to date, supplemented by a very accurate phenotypic characterization. The authors described four new unrelated probands carrying homozygous p.Arg69Alafs* mutations, each of them afflicted by severe DCM with a markedly early disease onset, ranging from congenital to 3 years old. In the same paper, they also reported the second instance of the homozygous *TNNI3* apparently synonymous variant, c.150G>A p.(Lys50=), which was demonstrated to lead to exon 4 skipping, resulting in a protein lacking 14 amino acids. The variant was identified in two siblings from remotely consanguineous healthy parents, presenting with slightly different phenotypes: the proband developed a low LVEF acute myocarditis triggered by parvovirus B19 infection, while her sister was diagnosed with severe DCM at the age of two years. Both of them required a heart transplant. The latest contribution to the genotypic spectrum of loss-of-function *TNNI3* variants has been made by Yu and colleagues [38], who reported a new synonymous variant, this time in position 24 of the cDNA sequence (c.24G>A, p.Ala8=), which was demonstrated to cause the retention of intron 2 resulting in a predicted premature stop codon. This variant was found in compound heterozygosis with a multi-exon deletion, in a patient who died of DCM at the age of 1 year. All the reported patients and their respective clinical, familial and molecular data are summarized in Table S1.

The patients diagnosed in our clinic shared many of the most prevalent characteristics described in previous reports. They both presented with DCM phenotype, which is the most represented in biallelic loss-of-function *TNNI3* carriers in the literature (16 out of 20 documented cases), sometimes in conjunction with LVNC, while RCMs and HCMs accounted for a minority of cases. They also confirm the early onset and the severe progression of these forms, and the almost constant requirement for heart transplantation within a short time after diagnosis. Notable exceptions in this regard are raised by the families reported by Murphy et al. and Pantou et al., which distinguished themselves both for a slower clinical course and, in the Pantou family, in remarkably different pathological findings, which are more reminiscent of the autosomal forms of *TNNI3*-related ICMs rather than the autosomal recessive ones. However, it should be noted that these two families shared a similar mutational landscape as well, both harboring homozygous *TNNI3* missense mutations instead of null mutations. As was already hinted by Carballo et al. [17] regarding the A2V variant, this kind of missense substitution may not lead to a complete loss of function of the protein as the nonsense, frameshift or splice variants have been demonstrated to, and thus may explain the different phenotypes. Further functional studies are warranted to better understand the mechanisms underlying this genotype–phenotype correlation.

Interestingly, the clinical presentation of our Patient A was associated with histopathological features of myocarditis in conjunction with viral infection, as described in the case studies of Belkaya and Seidel. However, these studies suffered from a selection bias in this respect, having both being identified from two distinct cohorts of myocarditis patients. It is nonetheless possible to hypothesize that viral infections in these patients may have played a role in triggering or anticipating the onset of dilated cardiomyopathy in *TNNI3* biallelic carriers. In this regard, a plausible association between acute myocarditis and ICMs in general has already been established by several observations in the literature [39,40,41,42]. However, it is not yet clear whether it is the inflammatory event that initiates the degenerative process, whether the structural impairment caused by ICM increases susceptibility to viral infections, or whether both of these mechanisms act synergistically by enhancing each other. Certainly, what can be derived from these observations is that the diagnosis of either a viral myocarditis or a genetically determined cardiomyopathy cannot be considered an exclusion criterion for the other, as was the case for our Patient A.

The variant p.(Arg98*) is described here for the first time in association with an autosomal recessive form of *TNNI3*-related dilated cardiomyopathy, despite the fact that this variant exhibits the highest relative carrier frequency in population databases (1/17,750 according to the gnomAD database). The frameshift variant p.Arg69Alafs* is the most common loss-of-function mutation reported in *TNNI3* homozygous DCM patients (9 out of 16 families), possibly because of the combination of a relatively high carrier frequency (1/26,222, second after p.Arg98*) and its occurrence in populations characterized by a higher degree of consanguinity.

The patients reported in this work serve as a further confirmation of the association between DCM2A phenotype and *TNNI3* biallelic loss-of-function mutations, consolidating the severe cardiac outcome shared by affected individuals with little to no variability, and the limited or even absent clinical impact of heterozygous variants on carriers. The non-negligible frequency of healthy carriers of *TNNI3* null mutations in the general population (48/100,000 individuals according to the gnomAD database) makes it desirable to improve general awareness regarding their reproductive risk.

## Data Availability

The data presented in this study are available on request to the corresponding author.

## Supplementary Materials

The following supporting information can be downloaded at: https://www.mdpi.com/article/10.3390/genes14030748/s1, Table S1: epidemiologic, phenotypic, and genotypic data of reported autosomal recessive *TNNI3*-patients.
